# An anterolateral papillary muscle rupture due to inferoposterior ischaemia

**DOI:** 10.1007/s12471-019-01355-3

**Published:** 2019-11-22

**Authors:** M. J. Schuuring, D. Robbers-Visser, A. H. G. Driessen, J. J. Piek

**Affiliations:** 1 Department of Cardiology, Amsterdam University Medical Centre, location AMC, Amsterdam, The Netherlands; 2 Department of Cardiology, Haga Teaching Hospital, The Hague, The Netherlands; 3 Department of Cardiothoracic Surgery, Amsterdam University Medical Centre, location AMC, Amsterdam, The Netherlands

**Fig. 1:**
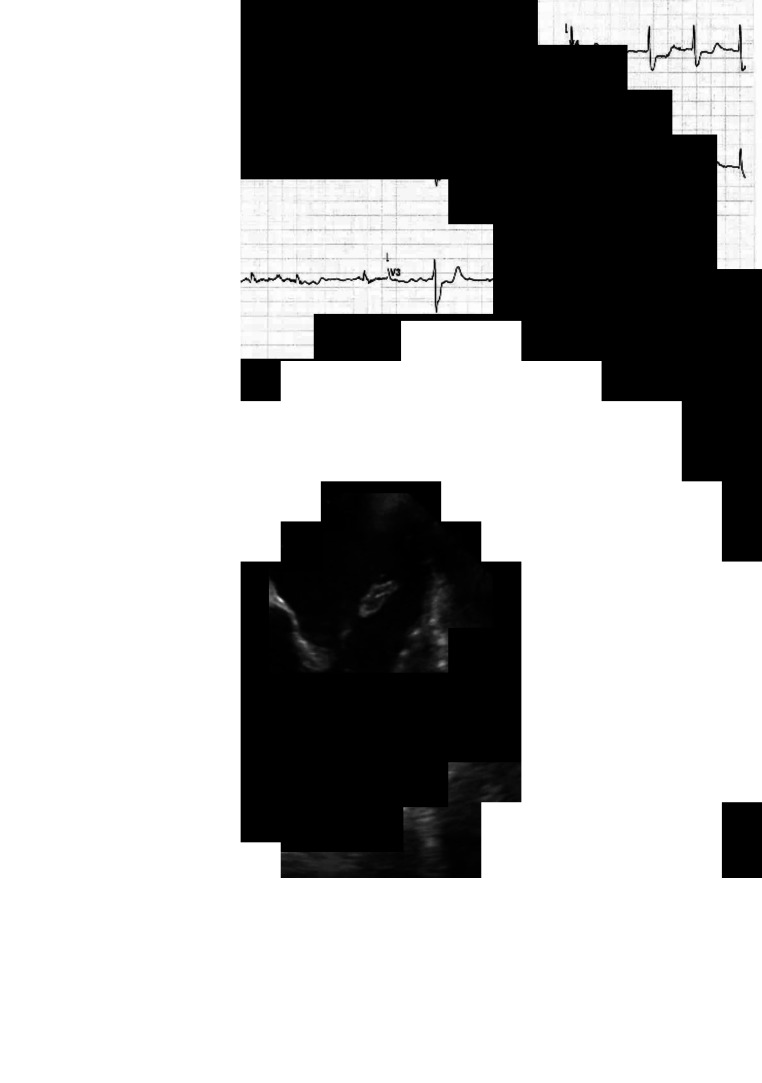
**a** Inferoposterior myocardial ischaemia. **b** The anterolateral papillary muscle rupture visualised on transoesophageal echocardiography

An 80-year-old man with a history of atrial fibrillation developed heart failure in 3 days. Soon after admission he went into cardiogenic shock. Electrocardiography demonstrated inferoposterior ischaemia (Fig. 1a). Transthoracic echocardiography demonstrated an anterolateral papillary muscle (APM) rupture, which was confirmed by transoesophageal echocardiography (Fig. 1b). An APM rupture was unexpected because of dual supply from the left anterior descending (LAD) and left circumflex (LCX) arteries [1–4]. The aetiology may be explained by the anatomy of the coronary circulation. Urgent angiography demonstrated an occlusion of the right coronary artery (RCA) with collateral vessels to the distal circumflex coronary (LCX) artery, a moderate distal left main stenosis, and both a subtotal LCX stenosis and a significant LAD lesion with collateral vessels to the RCA. Inferoposterior ischaemia likely induced coronary steal flow from the LAD, because the LAD stenosis was less severe than the RCA and LCX stenoses. Consequently, subendocardial ischaemia of the anterior wall emerged and led to an APM rupture. Urgent mitral valve replacement and concomitant coronary artery bypass grafting were performed. The haemodynamic parameters improved postoperatively.
